# Serum myo-inositol and valine improve metabolomic-based estimated glomerular filtration rate among kidney transplant recipients

**DOI:** 10.3389/fmed.2022.988989

**Published:** 2022-11-17

**Authors:** Jeffrey W. Meeusen, Frank Stämmler, Surendra Dasari, Eric Schiffer, John C. Lieske

**Affiliations:** ^1^Department of Laboratory Medicine and Pathology, Mayo Clinic, Rochester, MN, United States; ^2^Department of Research and Development, numares AG, Regensburg, Germany; ^3^Department of Quantitative Health Sciences, Division of Computational Biology, Mayo Clinic, Rochester, MN, United States; ^4^Department of Internal Medicine, Division of Nephrology and Hypertension, Mayo Clinic, Rochester, MN, United States

**Keywords:** magnetic resonance spectroscopy, creatinine, cystatin C, glomerular filtration rate, iothalamate clearance

## Abstract

**Background:**

Close monitoring of glomerular filtration rate (GFR) is essential for the management of patients post kidney transplantation. Measured GFR (mGFR), the gold standard, is not readily accessible in most centers. Furthermore, the performance of new estimated GFR (eGFR) equations based upon creatinine and/or cystatin C have not been validated in kidney transplant patients. Here we evaluate a recently published eGFR equation using cystatin C, creatinine, myo-inositol and valine as measured by nuclear magnetic resonance (eGFR_NMR_).

**Methods:**

Residual sera was obtained from a cohort of patients with clinically ordered iothalamate renal clearance mGFR (*n* = 602). Kidney transplant recipients accounted for 220 (37%) of participants.

**Results:**

Compared to mGFR, there was no significant bias for eGFRcr or eGFR_NMR_, while eGFRcr-cys significantly underestimated mGFR. P_30_ values were similar for all eGFR. P_15_ was significantly higher for eGFR_NMR_ compared to eGFRcr, while the P_15_ for eGFRcr-cys only improved among patients without a kidney transplant. Agreement with mGFR CKD stages of <15, 30, 45, 60, and 90 ml/min/1.73 m^2^ was identical for eGFRcr and eGFRcr-cys (61.8%, both cases) while eGFR_NMR_ was significantly higher (66.4%) among patients with a kidney transplant.

**Conclusion:**

The 2021 CKD-EPI eGFRcr and eGFRcr-cys have similar bias, P_15_, and agreement while eGFR_NMR_ more closely matched mGFR with the strongest improvement among kidney transplant recipients.

## Introduction

Serum creatinine-based estimated glomerular filtration rate (eGFR) is routinely used to detect and manage of kidney disease. Alternative eGFR methods using serum cystatin-C alone or in combination with serum creatinine have been developed and endorsed for use in patients when more accurate eGFR is required for clinical decision making (1, 2). The clinical utility of these equations has been independently validated (3). The general consensus is that in most clinical populations eGFR methods, which incorporate both creatinine and cystatin C outperform either in isolation (4–6). However, there are conflicting reports regarding the improvement in eGFR provided by cystatin C among patients with a kidney allograft (7, 8).

Recently, a race-free multi-marker eGFR method based on creatinine, cystatin C, valine and myo-inositol has been published (eGFR_NMR_) (9–11). The method was developed with the hypothesis that using multiple serum biomarkers would improve the eGFR performance. In order to simplify the measurement and quantify many different biomarkers simultaneously, the method used nuclear magnetic resonance spectroscopy (NMR). Preliminary studies evaluated dozens of metabolites associated with eGFR. The final model included serum valine, myo-inositol, creatinine, and cystatin C. We hypothesized that eGFR_NMR_ incorporating multiple biomarkers would improve eGFR performance in kidney transplant recipients. We also evaluated the newer race-free 2021 CKD-EPI eGFR equations in a cohort of kidney transplant recipients.

## Materials and methods

All patient data was accessed in compliance with the Mayo Clinic Institutional Review Board. Residual serum was obtained as available from all patients with a clinically ordered glomerular filtration rate (GFR) measured by iothalamate clearance at Mayo Clinic, Rochester, MN between April 2019 and March 2020 (*n* = 602). Indications for testing included post kidney transplant monitoring, CKD staging, and qualification of potential kidney donors. Patients who underwent GFR measurement for chemotherapy dosing, were paraplegic, quadriplegic, post amputation, or <18 years of age were excluded.

Glomerular filtration rate was measured by iothalamate clearance (non-radiolabeled). Patients were asked to report fasting to minimize dietary effects but well hydrated to stimulate urine flow. Following subcutaneous iothalamate administration oral hydration was continued and urine and plasma were collected in timed intervals for iothalamate quantification by liquid chromatography-tandem mass spectrometry (12). Body surface area as estimated by the DuBois formula and used to normalize GFR to 1.73 m^2^. Serum creatinine was measured using Roche Cobas clinical analyzers (c701 or c501; Roche Diagnostics; Indianapolis, IN, USA) via enzymatic methods standardized to international reference materials. Serum cystatin C was measured on a Roche Cobas c501 using an immunoturbidometric assay (Gentian; Moss, Norway) that was traceable to an international reference material.

Serum creatinine, valine and myo-inositol were measured by NMR spectroscopy as previously described (9). Samples were prepared by mixing of 540 μl serum and 60 μl of diluent. A total volume of 600 μl was filled into a 5 mm NMR-tube with a barcoded cap. Quality control samples were prepared by filling 600 μl of AXINON serum control 2.0 into a 5 mm NMR-tube (numares AG, Regensburg, Germany). Samples were pre-heated at 37°C for 7.5 min before NMR measurement in a Bruker Avance III 600 MHz and a 5 mm PATXI probe equipped with automatic Z gradients shimming. ^1^H-NMR spectra were recorded using a spectral width of 20 ppm, with a recycling delay of 1.5 s, 16 scans and a fixed receiver gain of 50.4. A cycling time d2 of 8 ms was used together with a corresponding T2 filter of 112 ms. Mixing time τ between two consecutive spin echoes was 400 μs. NMR data were automatically phase- and baseline-corrected using the lactate doublet at 1.32 ppm as reference. Metabolite quantification was automatically performed using curve-fitted pseudo-Voigt profiles.

Glomerular filtration rate was estimated by three different methods. The 2021 CKD-EPI eGFRcr was calculated using age, sex and serum creatinine measured by enzymatic assay (1). The 2021 CKD-EPI eGFRcr-cys was calculated using age, sex, serum creatinine measured by enzymatic assay and cystatin C measured by immunoassay (1). The eGFR_NMR_ was reported directly from Axinon software (numares, AG) which combines age, sex, cystatin C (immunoturbidometric) and NMR measured creatinine, valine and myo-inositol (10).

Bias was assessed as the median difference between measured and estimated GFR for a given category. The fraction of eGFR estimates within 30 and 15% of measured GFR were defined as P30 and P15, respectively. Agreement with measured GFR was determined by number of patients grouped into the following diagnostic categories <15, 15–29, 30–44, 45–59, 60–89, and ≥90 ml/min/1.73 m^2^ All calculations of performance evaluation and statistical tests were performed within R 4.0.2.

## Results

Kidney transplant recipients accounted for 220 (37%) of participants with a mean time post-transplant of 2.14 ± 3.2 years. There were no significant differences in age, sex, or BMI between patient groups with and without kidney transplant. Prevalence of diabetes and hypertension were significantly higher among kidney transplant recipients, as were serum concentrations of creatinine, cystatin C, valine and myo-inositol, while measured GFR was significantly lower (Table 1). Median serum myoinositol concentrations were significantly higher for kidney transplant recipients among patients with diabetes (85 vs. 73 μmol/L, *p* = 0.01) and without diabetes (68 vs. 59 μmol/L, *p* < 0.001).

**TABLE 1:** Demographic, clinical and laboratory findings among patients with a clinically ordered measured GFR test.

Characteristic	Kidney transplant	No kidney transplant	*P*-value
*N* (%)	220	382	
Age, year	55 ± 14	57 ± 13	n.s.
Female, *n* (%)	97 (44%)	170 (44%)	n.s.
Height	169 ± 11	171 ± 10	n.s.
Weight	86 ± 22	86 ± 21	n.s.
BMI, kg/m^2^	30 ± 6.1	29 ± 5.9	n.s.
Diabetes, *n* (%)	88 (40%)	70 (18%)	<0.01
Hypertension, *n* (%)	199 (90%)	144 (38%)	<0.001
Other Organ Transplant (not Kidney), *n* (%)	38 (17%)	113 (29%)	<0.01
Measured GFR, ml/min/1.73 m^2^	59 ± 20	74 ± 30	<0.01
Measured GFR group			
<15 ml/min/1.73 m^2^	1 (0.5%)	3 (0.8%)	n.s.
15–29 ml/min/1.73 m^2^	16 (7.3%)	25 (6.5%)	n.s.
30–44 ml/min/1.73 m^2^	43 (19.5%)	37 (9.7%)	<0.01
45–59 ml/min/1.73 m^2^	62 (28.2%)	74 (19.4%)	0.01
60– 89 ml/min/1.73 m^2^	86 (39.1%)	126 (33.0%)	n.s.
≥90 ml/min/1.73 m^2^	12 (5.5%)	117 (30.6%)	<0.01
Creatinine, mg/dl	1.40 ± 0.49	1.21 ± 0.57	<0.01
Cystatin C, mg/L	1.53 ± 0.55	1.31 ± 0.62	<0.01
Myo-inositol, μmol/L	78.3 ± 25.5	68.0 ± 03.3	<0.01
Valine, μmol/L	318 ± 71	298 ± 75	<0.01

**FIGURE 1:**
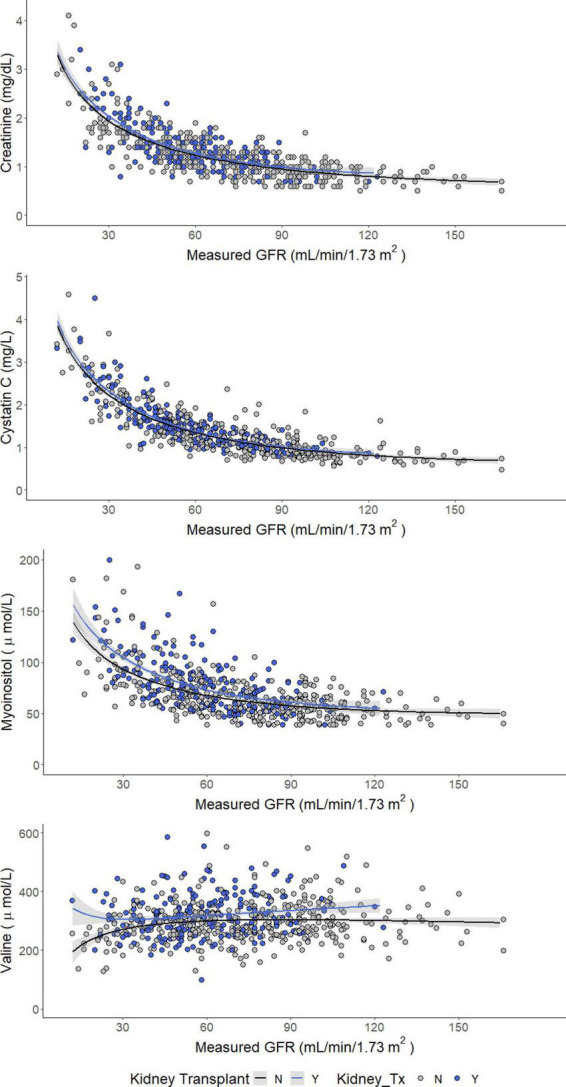
Concentrations of creatinine (enzymatic), cystatin C, myo-inositol, and valine as a function of measured GFR.

Concentrations of serum creatinine, cystatin C, and myo-inositol increased as GFR decreased, whereas serum valine decreased (Figure 1). Comparing patients with vs. without a kidney transplant found no difference in the relationship between measured GFR and serum concentrations of creatinine and cystatin C. However, the concentrations of serum valine were significantly higher among kidney transplant recipients with mGFR < 60 ml/min/1.73 m^2^ (median 300 μmol/L vs. 284 μmol/L, *p* < 0.05). Furthermore, serum myo-inositol was higher among kidney transplant recipients across the entire measured GFR range (median 73 vs. 62 μmol/L, *p* < 0.001).

**FIGURE 2:**
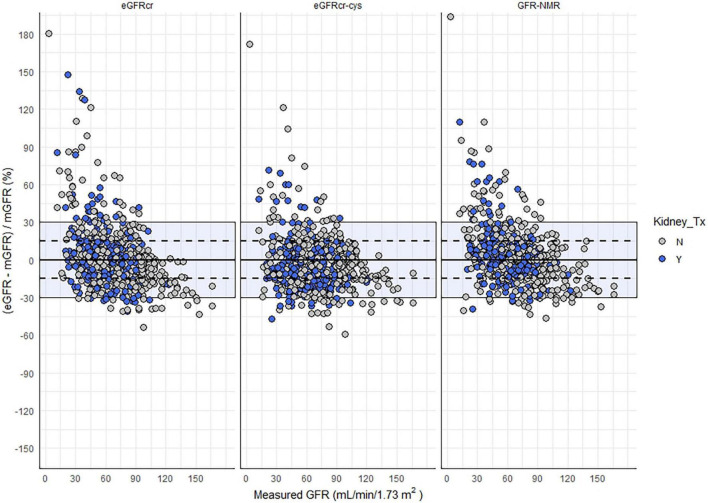
Relative difference between eGFR and mGFR as a function eGFR method and kidney transplant status. Shaded area indicates eGFR values within 30% of mGFR, and the dashed lines represent values within 15% of mGFR.

There was no significant bias, assessed as the median difference with measured GFR, among kidney transplant recipients by eGFRcr or eGFR_NMR_ (Table 2). However, eGFRcr-cys underestimated measured GFR by a slight but significant margin (−4 mL/min/1.73 m^2^, *p* < 0.05). The number of samples within 30% of measured GFR (P_30_) ranged between 85–90% and was not significantly different regardless of equation or kidney transplant status (Figure 2). However, significantly more kidney transplant recipients were within 15% of measured GFR (P_15_) using the eGFR_NMR_ compared to eGFRcr (67% vs. 57%, *p* = 0.03). When categorizing patients according to CKD diagnostic thresholds of <15, 15–29, 30–44, 45–59, 60–89, and ≥90 ml/min/1.73 m^2^, eGFR_NMR_ correctly classified significantly more kidney transplant recipients than eGFRcr and eGFRcr-cys (66% vs. 62%, *p* = 0.04).

**TABLE 2:** Method comparison for estimated versus measured GFR by eGFR equation and kidney transplant status.

	eGFR_cr_	eGFR_cr–cys_	*P*-value*	eGFR NMR	*P*-value*
Bias: median difference; ml/min/1.73 m^2^ (95% CI)					
Kidney transplant	−0.05 (−1.67 to 1.36)	−3.84 (−4.83 to −2.51)	<0.01	0.412 (−1.30 to 1.68)	0.82
No kidney transplant	−2.17 (−3.53 to −0.829)	−3.57 (−4.72 to −1.89)	0.04	−0.12 (−1.34 to 1.17)	0.96
P_15_; % (95% CI)					
Kidney transplant	57.3 (50.7–63.8)	60.9 (54.5–67.4)	0.43	67.3 (61.1–73.5)	0.03
No kidney transplant	48.7 (42.1–55.3)	56.0 (49.5–62.6)	0.04	58.6 (52.1–65.1)	0.005
P_30_; % (95% CI)					
Kidney transplant	85.0 (80.3–89.7)	90.0 (86–94)	0.11	90.9 (87.1–94.7)	0.06
No kidney transplant	84.8 (81.2–88.4)	86.9 (83.5–90.3)	0.41	85.3 (81.8–88.9)	0.84
Agreement;% (95% CI)					
Kidney transplant	61.8 (55.4–68.2)	61.8 (55.4–68.2)	1.0	66.4 (60.1–72.6)	0.04
No kidney transplant	58.6 (53.7–63.6)	61.8 (56.9–66.7)	0.04	60.7 (55.8–65.6)	0.55

*Denotes *p*-value comared to eGFRcr.

Using eGFRcr concordantly classified 82% (105 of 119) of kidney transplant recipients with measured GFR < 60 mL/min/1.73m^2^ and 78% (79 of 101) as >60 ml/min/1.73 m^2^. Applying the eGFRcr-cys equation correctly reclassified an additional 10.9% as <60 ml/min/1.73 m^2^, and incorrectly reclassified 2.8% of patients as >60 ml/min/1.73 m^2^ for a net reclassification improvement of 8.1% (Table 3). Reclassification according to eGFR_NMR_ would correctly reclassify 4.2% of kidney transplant recipients as <60 ml/min/1.73 m^2^, and correctly reclassify an additional 8.9% as >60 ml/min/1.73 m^2^, for a net reclassification improvement of 13.1%.

**TABLE 3:** Reclassification of patients into correct measured GFR clinical categories.

	Kidney transplant recipients		No kidney transplant	
CKD Reclassification*	eGFR_cr–cys_	eGFR_NMR_	eGFR_cr–cys_	eGFR_NMR_
<60 ml/min/1.73 m^2^	10.9% (5.3–16.5)	4.2% (0.6–7.8)	6.8% (2.5–11.1)	−1.5% (−3.6–0.6)
≥60 ml/min/1.73 m^2^	−2.8% (−6.3 to 0.3)	8.9% (3.4−14.5)	−2.8% (−6.0 to 0.4)	2.2% (1.0−5.4)
Net	8.1% (0.8−5.4)	13.1% (0.4−17.6)	5.6% (3.3−7.9)	1.7% (0.4–3.0)

*Relative increase or decrease in mGFR agreement compared to eGFRcr. Parentheses indicate 95th percentile confidence intervals. Confidence intervals that include zero are not significantly different than classification agreement using eGFRcr.

## Discussion

In this study we evaluated the performance eGFRcr and 2 multi-marker eGFR equations for predicting mGFR among patients with and without kidney transplant. Our findings confirm previous reports that including both creatinine and cystatin C (eGFRcr-cys) improves eGFR classification. The improvements for eGFRcr-cys were modest and the performance was consistent across all patients regardless of kidney transplant status.

Addition of two novel serum biomarkers, valine and myo-inositol along with creatinine and cystatin C (eGFR_NMR_) improved the P15 and concordance compared to eGFRcr and eGFRcr-cys. Previous studies have reported associations between kidney failure and increases in both myo-inositol and valine (13–16). In our cohort, the benefits of myo-inositol and valine in GFR estimation were stronger among kidney transplant recipients. Importantly, using eGFR_NMR_ improved agreement with CKD staging and net reclassification.

Previous studies have reported an association between myo-inositol and kidney disease progression (14, 16). Of specific interest are reports of altered inositol metabolism following kidney transplantation (17) and among patients taking calcineurin inhibitors commonly prescribed for immunosuppression following solid organ transplant (18). Teasing out the potentially confounding interactions between reduced kidney function, transplantation, immunosuppression and serum metabolites requires more study.

Elevated plasma valine is linked to metabolic disturbances and cardiovascular disease risk profiles in several non-transplant populations (19, 20). Hence, the increased valine concentrations observed in the transplant recipients of our cohort might point to a higher risk for CVD as indicated by the high prevalence of the conventional CVD risk factors, i.e., diabetes, hypertension, and by the drugs used for immunosuppression (21).

Some limitations of our study include the small number of participants with low measured GFR, which prevented analysis of potential impact on kidney transplant listing or re-listing using the multi-marker eGFR methods. While the differences between eGFR metrics were arguably marginal, they might be especially relevant for these use-cases. Nevertheless, the sample-size had sufficient statistical power to distinguish between method performance based on kidney transplant status.

NMR spectrometry is not-widely available in routine clinical laboratories; however, it is routinely used at reference laboratories for specialty lipid testing, which can guide cardiovascular and endocrinology care management. The improvement in eGFR by inclusion of multiple biomarkers suggests that NMR and its capability to quantify many analytes by a simultaneous measurement may be uniquely suited to investigate the metabolomics of kidney function. Further study to confirm these findings and investigate the relationship between kidney function and the serum concentrations of myo-inositol, valine and other potential biomarkers using NMR is warranted.

## Data availability statement

The raw data supporting the conclusions of this article will be made available by the authors, without undue reservation.

## Ethics statement

The studies involving human participants were reviewed and approved by Mayo Clinic Institutional Review Board. Written informed consent for participation was not required for this study in accordance with the national legislation and the institutional requirements.

## Author contributions

JM, FS, and SD participated in data acquisition and analysis. All authors contributed to drafting and editing of the manuscript.
